# A case report of exophytic nasal papilloma with acute dacryocystitis as the first symptom

**DOI:** 10.1186/s12886-021-02223-8

**Published:** 2021-12-29

**Authors:** Ya Mo, Danning Long, Luoxiang Li, Yanlin Zheng

**Affiliations:** 1 Department of Opthalmology, Hospital of Chengdu University of Traditional Chinese Medicine, No.39 Shi-er-qiao Road, Chengdu, 610072 Sichuan Province PR China; 2 Department of Ophthalmology, Chengdu University of Traditional Chinese Medicine, Chengdu, 610072 China; 3 Department of Pathology, Chengdu University of Traditional Chinese Medicine, Chengdu, 610072 China

**Keywords:** Acute dacryocystitis, Exophytic nasal papilloma, Nasal endoscopy, Dacryocystorhinostomy, Dacryocystectomy

## Abstract

**Background:**

This study aims to explore a case of exophytic nasal papilloma with acute dacryocystitis as the first symptom.

**Case presentation:**

A 72-year-old male patient complaining of “a 10-year history of tearing and purulent discharge from the right eye, with subsequent redness and pain in the inner canthus for three days” was initially diagnosed with acute dacryocystitis of the right eye. The patient was treated with anti-inflammatory therapy. However, the redness and swelling of the inner canthus continued to increase. An endoscopic dacryocystorhinostomy of the right eye was performed under general anesthesia. A large amount of purulent secretion was drained during the operation. As a result, the swelling of the inner canthus was significantly reduced. A routine intra-operative biopsy of the wall of the lacrimal sac revealed an exophytic nasal papilloma. A second biopsy, 1 week after the surgery, revealed the same result. The patient was advised to undergo a dacryocystectomy once the swelling had subsided. However, the patient was reluctant to undergo this surgery and remains under clinical observation.

**Conclusion:**

It is rare for an exophytic nasal papilloma, which is a benign tumor in the lacrimal sac, which has the potential for recurrence and malignant transformation, to manifest with acute dacryocystitis as the first symptom. Therefore, this case report could provide a reference for the future clinical diagnosis of this disease.

## Background

Acute dacryocystitis is characterized by purulent inflammation of the lacrimal sac that manifests as orbital cellulitis. With a lack of timely treatment, it can cause systemic issues such as irreversible vision loss [1] and cavernous sinus thrombosis [2]. Therefore, the etiology should be identified and the disease actively treated. Acute dacryocystitis mostly occurs as a result of the presence of chronic dacryocystitis and is correlated with the virulence of the invading bacteria and/or decreased resistance. The most common pathogen is *Staphylococcus aureus*. However, related reports of exophytic nasal papilloma in the lacrimal sac manifesting as acute dacryocystitis, accompanied by *Staphylococcus aureus* infection, are clinically rare. Although there have been clinical reports of dacryocystic papilloma [3], none have occurred with acute dacryocystitis. One such case report follows.

## Case presentation

### Clinical material

A 72-year-old male patient presented with “a 10-year history of tearing and purulent discharge from the right eye, with subsequent redness and pain in the medial.

canthus for 3 days.” Ten years previously, the patient had had no obvious cause for tearing and purulent discharge from the right eye, and it was not taken seriously. Three years ago, a right lacrimal duct placement was performed in another hospital (specifics unknown) but the patient still had tearing and purulent discharge after withdrawal of the tube. Four months previously, a localized bulge in the medial canthus of the right eye appeared, which the patient had ignored. Three days previously, the patient had suddenly developed swelling and pain in the inner canthus. He was diagnosed, in hospital, with acute dacryocystitis of the right eye. An intravenous infusion of drugs was started, but there was no obvious improvement. The details of this infusion are unknown. The redness and swelling gradually spread to the upper and lower sections of the orbit, with purulent secretions from the right eye.

Three days later, the patient was admitted to the hospital with a diagnosis of acute dacryocystitis of the right eye. On admission, the patient was experiencing difficulty opening the right eye, and there was obvious swelling of the lacrimal sac area and surrounding tissue, with unclear boundaries. The patient was also experiencing tenderness in this area. The patient did not have pyrexia and was not experiencing general weakness.

**Fig. 1:**
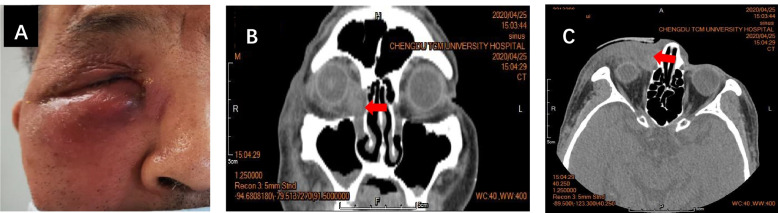
The patient’s eye appearance and results of the CT scan of the sinuses. **A** The patient’s condition at admission. Redness and swelling in the right lacrimal sac area are observably visible and without fluctuation. **B** The coronal CT image. A comparison between the left and right lacrimal sac areas; swelling of the soft tissue in the orbit and lacrimal sac areas of the right eye are clearly visible (as shown by the red arrow). **C** The horizontal CT image shows thickening of the soft tissue opacity in the right lacrimal sac and face area (as shown by the red arrow)

A coronavirus test was negative; blood test results revealed a white blood cell count of 10.72 × 10^9^ /L (normal range 3.5–9.5 × 10^9^ /L), neutrophils of 9.46 × 10^9^ /L (normal range 1.8–6.3 × 10^9^ /L), lymphocytes of 0.53 × 10^9^ /L (normal range 1.1–3.2 × 10^9^ /L), and C-reactive protein in whole blood of 33.07 mg/L (normal range 0–8 mg/L). There were no obvious abnormalities in the remaining indicators. The results of a CT scan of the sinuses were as follows: swelling of the soft tissue of the orbit, face, and the lacrimal sac area of the right eye and soft tissue opacity in the lacrimal sac area of the right side, the nature of which needed to be determined by further clinical examination; the nasal septum was shifted to the healthy side (Fig. 1). Upon completion of the auxiliary examinations, a systemic intravenous infusion of the anti-inflammatory supportive therapy cefuroxime sodium (cefuroxime sodium for injection, 1.5 g, Medochemie Ltd., production batch number C505B0) was started as follows: 0.9% NaCl/100 ml + cefuroxime sodium 1.5 g 30 gtt/min q12h.

### Treatment

This therapy was continued for 2 days (intravenous infusion of cefuroxime sodium 1.5 g + 0.9% NaCl injection 100 ml, once every 12 h) but failed to control the redness and inflammation; the area of swelling expanded.

After 2 days of treatment, blood tests were conducted once again. The results indicated a white blood cell count of 7.23 × 10^9^ /L (within the normal range), neutrophils of 6.11 × 10^9^ /L (within the normal range), lymphocytes of 0.5 × 10^9^ /L (a further decrease from the previous blood test), and C-reactive protein in whole blood of 58.43 mg/L (a further increase from the previous blood test). There were no obvious abnormalities in the remaining indicators.

**Fig. 2:**
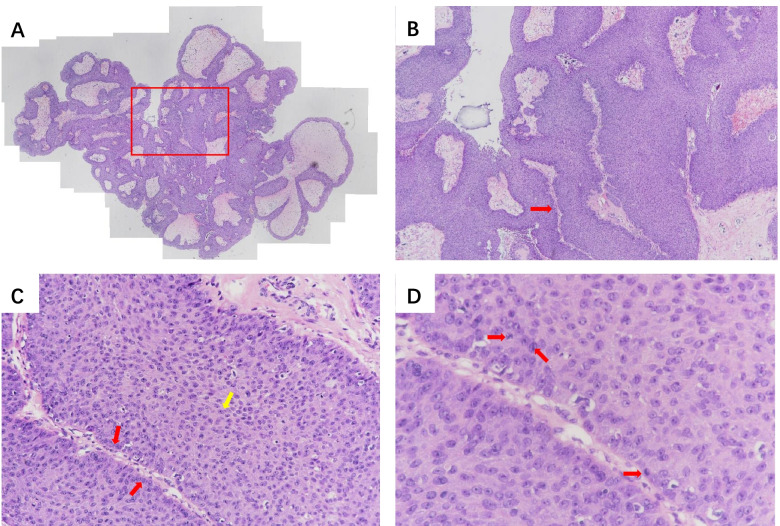
The pathological results of the exophytic nasal papilloma (hematoxylin and eosin staining). **A** The tissue section under a microscope, × 40. The tumor grew with the manifestation of the papillary mass and was covered by the squamous epithelium (the scope within the red box illustrates the specimen under key observation). **B** Enlarged × 40, an axis of fibrous connective tissue is visible in the center (as shown by the red arrow). **C** Enlarged × 200, the basal cells are visible in the fiber axis with a neat arrangement (as shown by the red arrow). The spinous cells are also visible with a disordered arrangement and mild dysplasia (as shown by the yellow arrow). **D** Enlarged × 400, it can be seen that the darker cells with connected nuclei are the nuclear divisions of the epithelial spinous cells (as shown by the red arrow)

Subsequently, an endoscopic dacryocystorhinostomy with the placement of a drainage tube for the right eye was performed under general anesthesia. On dissection of the lacrimal sac in the nasal cavity, a large amount of yellow and white purulent discharge was visible inside the sac; redness and swelling of the eye were significantly relieved. The pus was extracted via a puncture for bacterial culture and identification. The results showed that one pathogen was isolated, i.e., *Staphylococcus aureus*. The wall of the lacrimal sac was taken for a routine biopsy and the results revealed an exophytic nasal papilloma (Fig. 2). Owing to the high likelihood of postoperative recurrence and hyperplasia, the patient was informed that the papilloma should be treated with dacryocystectomy, the removal of the orbital mass, and the removal of the neoplasm through an incision in the nasal cavity, once the inflammation was controlled.

**Fig. 3:**
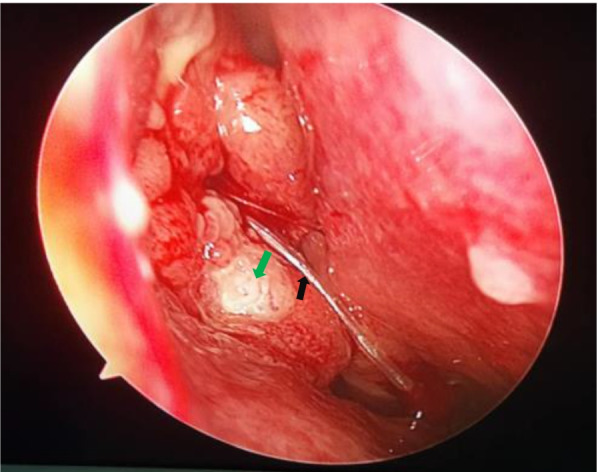
A white papillary neoplasm grew out of the nasal incision one week after the surgery. Note: the green arrow indicates the white papillary mass, and the black arrow indicates the drainage tube placed during surgery to facilitate the drainage of pus

Two days after surgery, blood tests were conducted once more and the results indicated a white blood cell count of 5.84 × 10^9^ /L (within the normal range), neutrophils of 4.5 × 10^9^ /L (within the normal range), lymphocytes of 0.82 × 10^9^ /L (an increase from the previous blood test), and C-reactive protein in whole blood of 38.95 mg/L (a decrease from the previous blood test). There were no obvious abnormalities in the remaining indicators. At the follow-up visit, 1 week after surgery, a new papillary neoplasm was visible growing from the nasal incision (Fig. 3). Again, a biopsy was performed, and the result revealed an exophytic nasal papilloma. It was recommended that once the inflammation had subsided, the patient should undergo dacryocystectomy, the removal of the orbital mass, and the removal of the neoplasm from the nasal cavity incision. However, the patient was reluctant to consent to the surgery and remains under clinical observation.

## Discussion and conclusions

Acute dacryocystitis most often occurs in the presence of chronic dacryocystitis and is correlated with the virulence of the invading bacteria and/or decreased bodily resistance. The most common pathogen is *Staphylococcus aureus* [4], which was present in this case. However, acute dacryocystitis with exophytic nasal caused by the pathogenic bacteria has not been reported. Acute dacryocystitis [5] is characterized by the purulent inflammation of the lacrimal sac; this can manifest as orbital cellulitis, typically involving the soft tissues of the orbital area, accompanied by localized redness and tenderness of the skin in the vicinity of the lacrimal sac. If the diagnosis and treatment of severe dacryocystitis are delayed, the orbital infection may cause permanent eye disease and irreversible vision loss [1] and can lead to systemic diseases such as cavernous sinus thrombosis [2]. If the effect of this anti-infective therapy is not satisfactory, acute-phase endoscopic dacryocystorhinostomy can quickly reduce inflammation, with a success rate of approximately 82.1% [6].

The patient presented to us with complaints of right-sided epiphora and purulent discharge of a 10-year duration, as well as swelling along the lacrimal sac area for 3 days, and was diagnosed with acute dacryocystitis. After systemic anti-inflammatory treatment, the redness and swelling of the right-sided lacrimal sac area continued to increase. Although the patient underwent CT examination, showing soft tissue images in the lacrimal sac area, surgery could not cut it from the skin due to acute facial inflammation. To prevent the inflammation from continuing, endoscopic dacryocystorhinostomy under nasal endoscopic surgery was performed. From the third day after the surgery, the pain and swelling of the patient’s lacrimal sac area were significantly relieved.

An exophytic nasal papilloma was found during a routine lacrimal sac biopsy; a study of a total of 3865 histopathologically examined lacrimal sac wall biopsy specimens from 3662 patients showed that lacrimal sac-specific pathologies were present in 226 cases (5.85%). Among them, 24.34% of cases were found to be benign lesions of the lacrimal sac [7]. This case report belongs to benign pathology from lacrimal sac wall as the same.

The nasal cavity and paranasal sinuses are important parts of the upper airway in which many types of tumors can occur. Respiratory papillomatosis is a virus-derived disease that may affect the larynx, trachea, and lower respiratory tract. Approximately 5% of these benign and locally aggressive tumors are related to squamous cell carcinoma [8]. According to the classification of upper respiratory tract tumors issued by the World Health Organization (1991, 2nd ed.), nasal papillomas are divided into three histopathological types, i.e., exophytic, inverted, and columnar cell papillomas. At the histological level, all three types have the characteristics of a thickened epithelium and the spread of mucosal cells or cysts [9].

Ward first recorded the occurrence of papilloma in the sinus cavity in 1854 [10]. It occurred in the Schneiderian mucosa and was a benign tumor with a likelihood of recurrence and malignant potential. Exophytic nasal papillomas account for approximately 0.4–4.7% of nasal tumors, with a low malignant transformation rate. From a macro perspective, they are usually milky white papillomas. From a micro perspective, they comprise papillary lobes with a fibrovascular core [11].

In terms of treatment, there is insufficient evidence to support the efficacy of antiviral drugs as a therapy in the management of recurrent respiratory papillomatosis in both children and adults [12]. Clinically, surgical resection is the generally adopted therapy. Owing to advancements in surgical techniques and the aesthetic requirements of patients, exophytic papillomas originating from the nasal cavity are most often operated on using nasal endoscopy. The field of vision in this surgical procedure is larger and clearer, and the tumor can be accurately located, reducing the incidence of postoperative complications and improving the rate of tumor control. Long-term, consistent follow-up with patients after surgery is required for the early detection of recurrence, which should be treated surgically because it may be correlated with malignancy [13]. However, in addition to endoscopic surgery, treatment must be closely coordinated with the clinic. In the present case, the exophytic papillomas involved the lacrimal sac and the surrounding orbit. Therefore, it was difficult to remove the tumor via nasal endoscopic surgery alone. The lacrimal sac and the surrounding orbital neoplasm needed to be removed from the orbital lacrimal sac area.

In this case, the patient was admitted to the hospital with acute dacryocystitis as the first symptom. At the time of admission, the white blood cells, neutrophils, and C-reactive protein in whole blood were all increased, and the number of lymphocytes was decreased, suggesting acute bacterial infection. After 2 days of administering cefuroxime sodium as a systemic anti-inflammatory and supportive therapy, the white blood cell and the neutrophil count had reduced to normal, and the number of lymphocytes was slightly lower than before, suggesting a reduction in inflammatory response. The C-reactive protein in whole blood was increased when compared with the levels 2 days previously, suggesting that the bacterial infection had increased. In this case, endoscopic dacryocystorhinostomy was performed because systemic anti-inflammatory treatment could not control the inflammation. After the treatment, the redness and swelling in the lacrimal sac area decreased significantly, and the white blood cell and neutrophil levels returned to normal. The number of lymphocytes rose to normal, and the C-reactive protein in whole blood decreased significantly compared with the previous blood test results. This indicated a reduction in the inflammation and signaled that the therapy had been effective. The wall of the lacrimal sac was removed for routine biopsy, the result of which was exophytic nasal papilloma. The bacterial culture of the pus extracted from the puncture showed an increase in *Staphylococcus aureus*, which is the most common gram-positive bacterium. In this case, the infection with *Staphylococcus aureus* was mainly related to chronic dacryocystitis. Simultaneously, the exophytic papilloma grew in the limited space of the lacrimal sac area, resulting in the compression of the lacrimal duct, poor discharge of tears, and *Staphylococcus aureus* retention. Eventually cause acute dacryocystitis.

In this case, the lacrimal sac was the primary site of occurrence, and the papilloma grew into the nasal cavity after the endoscopic dacryocystorhinostomy due to acute dacryocystitis. If a second surgical resection cannot be completely performed using an endoscope, it should be conducted under direct vision to remove the lacrimal sac and surrounding orbital masses. The combination of the two operations can completely remove the masses to minimize the chance of recurrence; however, the possibility of postoperative tearing is high, and the possibility of recurrence cannot be ruled out. Nevertheless, because the patient was unwilling to undergo this treatment approach, the case remains under clinical observation.

In summary, primary exophytic nasal papillomas of the lacrimal sac are clinically rare, with only a handful of case reports on inverted papilloma of the lacrimal sac present in the literature [3, 14]; furthermore, exogenous papilloma in the lacrimal sac manifesting with acute dacryocystitis as the first symptom has not yet been reported. Therefore, this case report provides a reference for future clinical diagnoses of this disease.

## Data Availability

The datasets used and/or analysed during the current study available from the corresponding author on reasonable request.

## References

[1] Lwry EA, Kalin-Hajdu E, Kersten RC, Vagefi MR (2018). Acute vision loss from Dacryocystitis. JAMA Ophthalmol.

[2] Mansour AM, Kheir-Jurdi W, Hadi UE, Awar G (2017). Odontogenic abscess mimicking acute dacryocystitis. BMJ Case Rep.

[3] Cheang YFA, Loke D (2020). Inverted papilloma of the lacrimal sac and nasolacrimal duct: a case report and review of the literature. Cureus..

[4] Mills DM, Bodman MG, Meyer DR, Morton AD (2007). 3rd; ASOPRS Dacryocystitis study group. The microbiologic spectrum of dacryocystitis: a national study of acute versus chronic infection. *Ophthalmic*. Plast Reconstr Surg.

[5] Li EY, Wong ES, Wong AC, Yuen HK (2017). Primary vs secondary endoscopic Dacryocystorhinostomy for acute Dacryocystitis with lacrimal sac abscess formation: a randomized clinical trial. JAMA Ophthalmol..

[6] Pakdel F, Soleimani M, Kasaei A, Ameli K, Pirmarzdashti N, Tari AS, Ghasempour M, Banafsheafshan A (2020). Shifting to very early endoscopic DCR in acute suppurative dacryocystitis. Eye (Lond).

[7] Koturovi Z, Kneevi M, Rai DM (2017). Clinical significance of routine lacrimal sac biopsy during dacryocystorhinostomy: a comprehensive review of literature [J]. Bosn J Basic Med.

[8] Nomura K, Ogawa T, Sugawara M, Honkura Y, Oshima H, Arakawa K, Oshima T, Katori Y (2013). Association between septal deviation and sinonasal papilloma. Tohoku J Exp Med.

[9] Bishop JA (2017). OSPs and ESPs and ISPs, oh my! An update on Sinonasal (Schneiderian) Papillomas. Head Neck Pathol.

[10] Vorasubin N, Vira D, Suh JD, Bhuta S, Wang MB (2013). Schneiderian papillomas: comparative review of exophytic, oncocytic, and inverted types. Am J Rhinol Allergy.

[11] Glâtre R, De Kermadec H, Alsamad IA, Badoual C, Gauthier A, Brugel L, Parra C, Coste A, Prulière-Escabasse V, Bequignon E (2018). Exophytic sinonasal papillomas and nasal florid papillomatosis: a retrospective study. Head Neck.

[12] Chadha NK, James A (2012). Adjuvant antiviral therapy for recurrent respiratory papillomatosis. Cochrane Database Syst Rev.

[13] Wormald PJ, Ooi E, van Hasselt CA, Nair S (2003). Endoscopic removal of sinonasal invertedpapilloma including endoscopic medial maxillectomy. Laryngoscope..

[14] Purser J, Arffa R, Clark D (2019). Sinonasal (Schneiderian) papilloma of the lacrimal sac [J]. Baylor Univ Med Center Proc.

